# Asleep Speech Mapping Using Orofacial Muscles as Surrogates for Motor Speech in Patients Who Cannot Tolerate Awake Surgery: A Case Series

**DOI:** 10.7759/cureus.15861

**Published:** 2021-06-23

**Authors:** David Bonda, Justin W Silverstein, Joshua Katz, Jason A Ellis, John Boockvar, Randy D'Amico

**Affiliations:** 1 Neurosurgery, Lenox Hill Hospital/Donald and Barbara Zucker School of Medicine at Hofstra, New York, USA; 2 Neurology, Lenox Hill Hospital Northwell Health, New York, USA; 3 Neurology, Neuro Protective Solutions, New York, USA; 4 Neurosurgery, Lenox Hill Hospital/Donald and Barbara Zucker School of Medicine, New York, USA; 5 Neurosurgery, Lenox Hill Hospital Northwell Health, New York, USA; 6 Neurological Surgery, Northwell Health, New York, USA

**Keywords:** asleep speech, corticobulbar motor evoked potentials, cortical mapping, orofacial mapping, penfield method

## Abstract

Background

Bi-polar electrical cortical stimulation during awake craniotomy has been the gold standard for mapping eloquent cortex to preserve speech. Unfortunately, not all patients can tolerate awake surgery. Monopolar hi-frequency electrical stimulation can be conducted while a patient is under general anesthesia. Utilizing this technique and targeting the orofacial muscles as surrogates for motor speech may provide a limited alternative to awake cortical mapping in patients unable to undergo surgery awake.

Objective

To evaluate the utility of asleep motor speech mapping during dominant hemisphere craniotomy for lesion resection in patients who cannot tolerate awake surgery.

Methods

We describe a series of seven patients who underwent craniotomy for resection of intra-axial lesion in eloquent cortex for whom a novel “asleep speech” cortical stimulation paradigm was used for motor speech preservation.

Results

Compound muscle action potentials (CMAPs) from orofacial muscles involved in motor speech were recorded during direct cortical stimulation of eloquent cortex prior to and during lesion resection. Planned resections proceeded in all cases with no adverse neuromonitoring events. Speech was preserved in all patients.

Conclusions

To preserve motor speech functionality in patients unable to tolerate awake speech mapping, we employed a technique in which asleep neurophysiological mapping is specifically applied to motor cortex controlling the orofacial muscles of phonation and articulation. Further study is necessary regarding the safety and efficacy of this technique for motor speech preservation when awake surgery cannot be performed.

## Introduction

Awake cortical mapping using directly applied electric current has been the gold standard in eloquent cortex preservation during brain tumor and epilepsy surgery since its description by Penfield and Boldrey in 1937 [1-10]. While improvements in cortical motor stimulation and anesthetic technique have broadened the utility of awake craniotomies, there remain limitations to their applicability. Specifically, some patients are unable to tolerate awake brain surgery due to severe psychiatric disease, altered mental status, cognitive/developmental delay, comorbidities that do not permit conscious sedation, mixed/receptive aphasia, or age [11]. Language barriers may also rarely preclude the use of awake speech mapping [12]. Unfortunately, in these circumstances, there are few options available to neurosurgeons that can ensure the level of eloquent cortex preservation that awake surgery provides. 

Direct cortical mapping of motor cortex involving orofacial musculature as a surrogate for motor speech functionality was first described by Deletis et al [13]. Lenox Hill Northwell IRB approved this study and that consent was not necessary based on the evaluation. Here we present a clinical extension of their proof of concept with our technique for asleep motor speech preservation in a series of seven patients undergoing dominant-hemisphere craniotomy for lesional resection.

This technique involves stimulation of corresponding cortex and recording of compound muscle action potentials (CMAPs) from associated orofacial muscles to identify either the lateral motor cortex (M1) or the inferior frontal gyrus. This permits the surgeon to avoid the motor speech areas as described by Deletis et al. Our results suggest this technique to be safe and possible, though the limited alternative to awake cortical mapping in patients needing surgery adjacent to motor speech areas who cannot tolerate awake surgery.

## Materials and methods

Methods

This study was approved by the Institutional Review Board (IRB) at Lenox Hill Hospital. The medical records and surgical neurophysiology data from seven consecutive, dominant-hemisphere craniotomies for lesional resection were retrospectively analyzed. Three neurological surgeons from a single-center performed all the craniotomies with the patients under general anesthesia. Multi-modal neurophysiological monitoring and mapping was performed intraoperatively by a board-certified doctorate-level surgical neurophysiologist according to the protocol described below. 

The investigators reviewed the following: patient age, sex, preoperative diagnosis, preoperative presentation and neurologic exam, past medical history, location of lesion, contraindication for awake surgery, neurophysiological monitoring data, initial postoperative notes, first postoperative follow-up, most recent follow-up, and treatment course.

Operative techniques

Patients underwent general endotracheal anesthesia. Prior to patient positioning, neuromonitoring electrodes were placed for the various modalities applied. Patients were positioned by the surgical team and the planned craniotomy was performed in standard fashion. Cortical mapping techniques are described below. Corticectomies were planned based on the preservation of areas corresponding to motor speech [3,9,14-18].

Neurophysiological monitoring and mapping

A multi-modal neuromonitoring paradigm was employed with upper and lower extremity somatosensory evoked potentials (SSEP) (ulnar, median, and posterior tibial nerves), transcranial motor evoked potentials with limb recordings (TCMEP-L), corticobulbar motor evoked potentials (co-bulb MEP), electroencephalography (EEG), phase reversal technique (PRT), and direct cortical stimulation.

Orofacial mapping

Sensorimotor localization was conducted using PRT with median nerve stimulation and signal acquisition from a 1x6 subdural strip electrode straddled over the central sulcus. Once the Rolandic fissure was identified with PRT, the same 1x6 strip electrode was plugged into an ES-IX constant current stimulator (Cadwell Labs, Kennewick, WA). Using the Cascade Pro software (Cadwell Labs, Kennewick, WA), multi-pulse motor stimulation was independently delivered through each contact identified as over motor cortex via PRT. Stimulation confirmed motor cortex. A multi-pulse train of 5 with a 500µS pulse width and ISI of 2ms was used. Stimulation started at 15mA. If we obtained a CMAP from any orofacial muscle, we reduced the train pulses to 1 to ensure we were activating centrally and not peripherally [19]. Once the most robust motor response was obtained from the subdural electrode, the stimulation was reduced to submaximal stimulation, and the electrode was left in place to continuously monitor corticospinal and corticobulbar tract integrity for the remainder of the procedure. The same stimulation settings were applied using a handheld monopolar probe to stimulate the areas for planned corticectomy. Once the motor cortex was thresholded to submaximal stimulation, the stimulus intensity was increased by 1mA to reduce a possible false negative when stimulating around the corticectomy site.

Orofacial co-bulb and direct cortical stimulation (DCS) coverage

**Figure 1:**
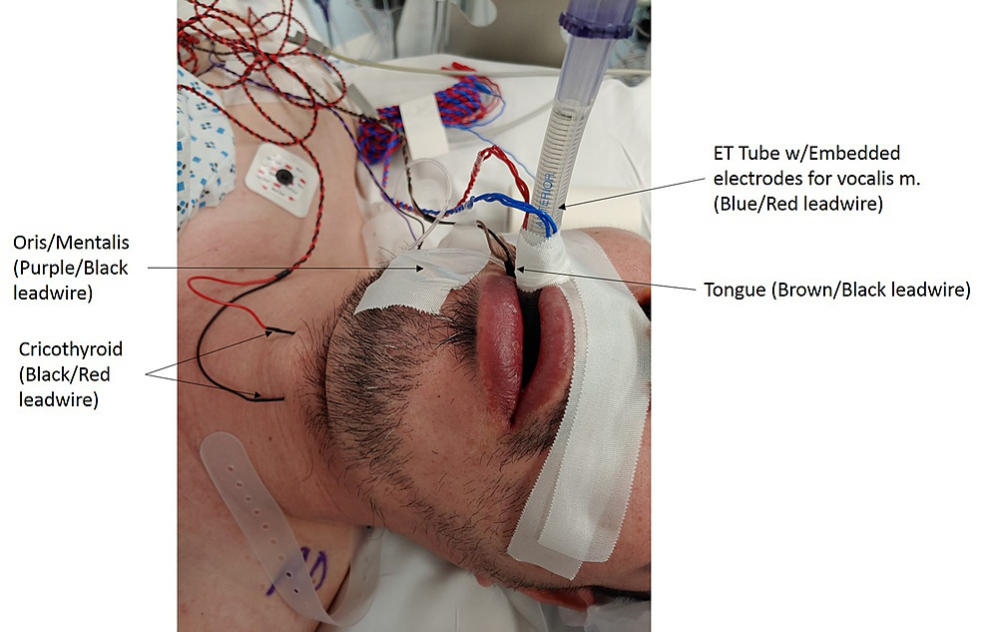
Setup of orofacial target muscles with standard neuromonitoring EMG electrodes. Referential recording montages are used for the bilateral cricothyroid muscles, as well as the oris and mentalis muscles on the right. A bipolar montage is used to target the tongue and the ET tube has embedded bipolar electrodes to record from the left and right vocalis muscles. EMG: electromyography; ET: endotracheal.

**Figure 2:**
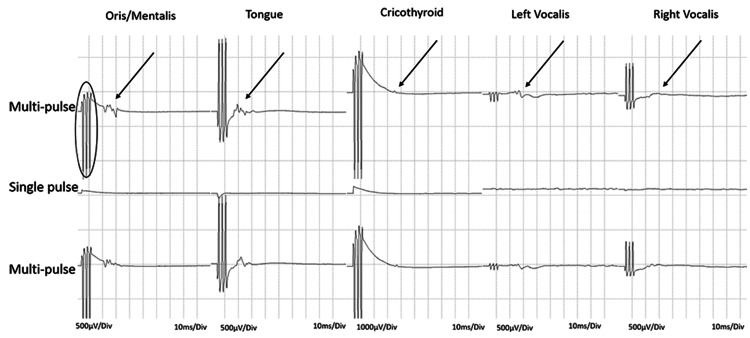
CMAPs acquired from the orofacial muscles by multi-pulse (circle) direct cortical motor stimulation (arrows). Note the disappearance of the CMAP when single-pulse stimulation is applied. This indicates the orofacial recordings are corticobulbar mediated vs peripheral activation. CMAPs: compound muscle action potentials.

We used four different muscle groups as targets for Co-bulb MEP and DCS to identify the orofacial area of the motor homunculus. Using an endotracheal tube with an embedded electrode allowed us to record from the bilateral vocalis muscles. Using standard intraoperative EMG needles, we also targeted the bilateral cricothyroid muscles, the contralateral oris/mentalis muscles, and the contralateral tongue (Figures 1, 2).

## Results

**Figure 3:**
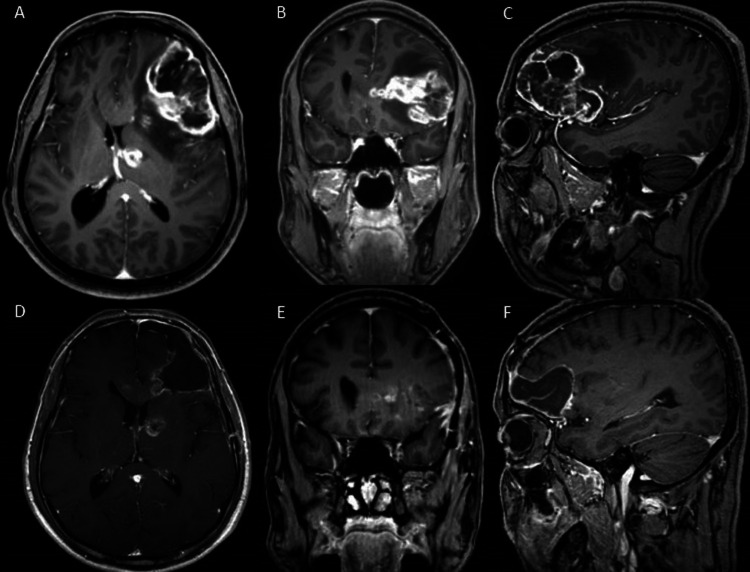
Pre- (A, B, C) and post-operative (D, E, F) images from patient #7 demonstrating enhancing, left frontal lesion abutting caudal inferior frontal operculum with edema extending into motor cortex. Use of direct cortical stimulation along these regions enabled CMAP acquisition prior to resection, and consequent sparing of regions controlling motor speech. CMAP: compound muscle action potential.

The use of asleep speech motor mapping was at the discretion of the attending neurosurgeon. All patients had dominant hemisphere lesions. Lesions were either adjacent to canonical language areas or the planned surgical approach risked injury to these areas (see Figure 3 for example case). All patients had contraindications to awake craniotomy (Table 1). Following craniotomy, the surgeon used navigation and standard anatomic landmarks to identify the area believed responsible for motor speech functionality. Positive mapping was performed using a flush tip monopolar stimulating probe at 1mA above submaximal stimulation to stimulate the cortical surface. Positive CMAPs were obtained from orofacial muscles in all patients excluding one. Patient #6 did not elicit any CMAPs from direct monopolar probe stimulation consistent with the anatomy of the lesion. In this specific case, the surgeon used the DCS to negative map this patient to confirm the safety of the planned corticectomy and trajectory for resection.

**Table 1:** : Patient characteristics. CMAP: compound muscle action potentials; GBM: glioblastoma multiforme; POD: post-operative day.

Patient	Age (years)	Lesion location	Diagnosis	Contraindication for awake surgery	Orofacial muscles identified	Initial follow up	1^st^ follow up	2^nd^ follow up
1	58	Left insula	GBM (Grade IV)	Positioning for approach	Vocalis and tongue	Intact	Intact (POD#11)	Intact
2	46	Left temporal	Low grade infiltrative glioma (IDH mutant)	Uncooperative/agitated	Mentalis/oris and cricothyroid	Intact	Intact (POD#27)	Intact
3	58	Left parietal	Metastatic pulmonary neuroendocrine carcinoma	Mental status decline	Vocalis	Intact	Intact (POD#19	Right hand/arm 4/5
4	79	Left posterior temporal	Metastatic renal cell carcinoma	Cognitive dysfunction/mental status decline	Vocalis	Intact	Intact (POD#29)	Intact
5	65	Left posterior temporo-occipital	Metastatic breast carcinoma	Seizures/status epilepticus	Vocalis	Intact	Intact (POD#12)	Intact
6	20	Left frontal	Cavernous malformation	Hemorrhagic lesion	No CMAP acquired	Intact	Intact (POD#10)	Intact
7	52	Left anterior inferior frontal	GBM (Grade IV)	Language barrier (Mandarin)	Face/vocalis/tongue	Intact	Intact (POD#13)	Intact

**Figure 4:**
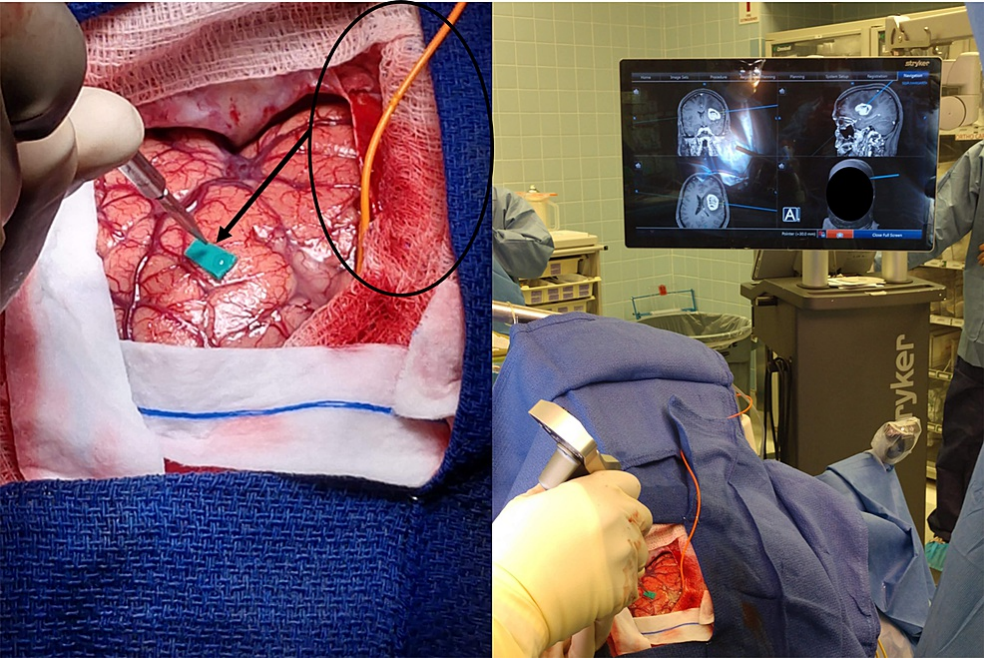
Marker placed by surgeon on area that activated orofacial muscles to direct cortical motor stimulation (arrow). Circle is lead wire for strip electrode to conduct functional continuous motor monitoring throughout the procedure.

For the six cases where orofacial CMAPs were obtained, a marker was placed over the area of the cortex that elicited responses (Figure 4). A corticectomy was then planned away from this area. The site of the planned corticectomy was subsequently stimulated and inactivation of orofacial muscles was confirmed. No response was elicited from any planned corticectomy site.

Planned resections proceeded in all cases with no adverse neuromonitoring events. Each patient awoke neurologically intact with no new-onset neurological or speech pathology. No patients demonstrated speech deficits or deterioration over three consecutive follow-up appointments. However, two out of six had new neurological symptoms at their most recent follow-up (33%), with patient #1 being alert and oriented x3 with weaker lower extremity muscles, and patient #3 having 4/5 strength in their right upper extremity. These were believed to be the result of disease progression and treatment effect in eloquent regions (Table 1). 

## Discussion

The Penfield method of awake cortical stimulation-based speech mapping involves stimulation of the cortical regions with a bipolar probe for 4 to 5 seconds using a long train of 50Hz or 60 Hz while the patient is asked to perform speech-requiring tasks [6,8,15,20-22]. The presence of speech arrest during stimulation is used to identify and preserve language cortex as well as inform the surgeon of functional boundaries to be preserved during resection. Subcortical stimulation has recently been added to this paradigm to ensure continuity of projection fibers from the cortical surface [7,23]. These techniques have allowed neurosurgeons to maximize lesion resection while minimizing post-operative speech deficits for over 60 years.

Unfortunately, not all patients who present with lesions in or near their speech area can tolerate brain surgery while awake. Furthermore, some centers that have standard neuro-monitoring capabilities may not be comfortable or equipped to handle awake craniotomies. We present a technique that may enable a surgeon preserve motor speech functionality in a population of patients without the need for awake speech mapping.

We employed a technique in which neurophysiological mapping is specifically applied to cortical regions controlling the orofacial muscles of phonation, in addition to standard asleep motor function mapping. These regions were originally identified by Deletis et al. in 2014 by comparing orofacial muscle latency responses elicited without speech production from the same areas that produced speech arrest [13]. Specifically, activation of the caudal opercular part of the inferior frontal gyrus corresponded with a long latency response (LLR) from the cricothyroid muscles, whereas activation of the lateral part of M1 (primary motor cortex) corresponded with a short latency response (SLR) from the laryngeal muscles. Both of these areas thus contribute to motor speech when elicited with the Taniguchi method of stimulation (5 pulse, short-train, high frequency, stimulation) [24]. These results helped Deletis et al. (2014) identify the lateral M1 and pars opercularis as anatomic landmarks contributing to motor speech.

We applied this technique for the first time in asleep patients during dominant-hemisphere lesion resection as a means of identifying and sparing cortical regions governing motor speech production during surgery. Lateral M1 was successfully identified in all six patients, and the caudal opercular part of the inferior frontal gyrus in one patient (Patient #7). Consistent with the original description, we were able to use these regions as a surrogate for motor speech and successfully avoided language disruption in all patients during lesion resection. The lesions resected included those adjacent to, or within, speech motor areas, as well as deep-seated lesions whose resection would potentially threaten motor speech functionality via surgical approach.

Motor language injury was avoided in all patients included despite a significant risk of such given the presence of each dominant hemisphere lesion. Because resection of each lesion proceeded without any adverse neuromonitoring deterioration to the orofacial musculature, we cannot definitively say that this technique prevented an otherwise inevitable language deficit. However, our successful mapping of the cortical regions corresponding to each orofacial muscle suggests this technique may be a viable alternative to awake speech mapping in patients deemed inappropriate for awake language mapping.

Given the complexity of the language system, awake craniotomy with intraoperative speech mapping remains the gold standard for attempting to achieve functional preservation during surgical resection of lesions arising within or adjacent to eloquent cortex. This is particularly true for lesions involving multiple contributing components of language such as the dorsal and ventral stream or area 55b. However, for lesions isolated to the inferior frontal gyrus or M1 arising in patients for which awake craniotomy is not practical or feasible, the technique we describe may be of benefit to identify the areas responsible for motor speech function in the asleep patient.

The limitations to this technique should be appreciated and we do not recommend this technique when awake surgery is feasible. The language motor system is complex and does not involve only the lateral M1 region and pars opercularis. Furthermore, this technique is primarily mapping corticobulbar white matter tracts. As a result, this technique cannot be conducted for lesions in the receptive or connective speech areas and cannot monitor higher function language components such as repetition, noun/verb, and or counting as these require higher-level function. In addition, the number of patients included remains small and the lesion locations heterogeneous. Most notably, there were no loss of signals or potentials that correlated to language deficits as regions of cortex that demonstrate positive stimulation were avoided.

Regardless, we present these data as a support of the concept of asleep motor speech mapping and propose that the technique may provide a reliable alternative to awake speech mapping in patients deemed inappropriate for awake surgery or in centers without the resources or experience to perform awake language mapping. A prospective cohort and larger sample size are needed to fully determine the safety and efficacy of this technique.

## Conclusions

Asleep neurophysiologic identification of the orofacial area of the motor homunculus may be a safe though limited alternative to awake craniotomy for patients with lesions abutting the posterior opercular region and lateral M1 region who cannot tolerate standard awake craniotomies with intraoperative speech mapping. Further research into its safety and utility is warranted
